# NADPH Oxidase 1 Mediates Endothelial Dysfunction and Hypertension in a Murine Model of Obesity

**DOI:** 10.3390/antiox15010060

**Published:** 2026-01-01

**Authors:** Caleb A. Padgett, Joshua T. Butcher, Sebastian Larion, James D. Mintz, David J. R. Fulton, David W. Stepp

**Affiliations:** 1 Vascular Biology Center, Medical College of Georgia, Augusta University, Augusta, GA 30912, USA; capadgett@augusta.edu (C.A.P.); slarion@gmail.com (S.L.); jmintz@augusta.edu (J.D.M.); dfulton@augusta.edu (D.J.R.F.); 2 Department of Physiological Sciences, Oklahoma State University, Stillwater, OK 74078, USA; joshua.butcher@okstate.edu

**Keywords:** NADPH oxidase, metabolism, reactive oxygen species, physiology, vascular biology

## Abstract

Obesity is a foremost risk factor for the development of cardiovascular disease, a hallmark of which is chronic vascular inflammation and overproduction of reactive oxygen species (ROS). NADPH oxidases (NOX) are central mediators of ROS overproduction in the obese vasculature, yet a complete understanding of the mechanism underlying their dysregulation in obesity remains poorly understood. Herein, we investigated the contribution of NOX1 in obesity-associated hypertension and evaluated the therapeutic potential of pharmacologically targeting NOX1 using the novel inhibitor GKT771. In obese *db*/*db* mice, NOX1 deletion ameliorated hypertension independent of metabolic improvements such as weight loss or improved glucose handling. Furthermore, NOX1 deletion improved renal sodium handling with no compensatory upregulation of other NOX isoforms. Importantly, treatment with the NOX1-specific inhibitor GKT771 rescued endothelial function in obese mice, restoring microvascular function to levels observed in lean controls. These data highlight the importance of NOX1 as a driver of endothelial dysfunction in obesity and suggest that NOX1 inhibition may offer a novel therapeutic strategy for obesity-associated endothelial dysfunction and its downstream cardiovascular complications.

## 1. Introduction

Within the United States, obesity remains one of the greatest drivers of mortality, resulting in over 700,000 excess deaths yearly and contributing to over $250 billion in healthcare costs [1]. As of 2024, over 70% of Americans are overweight or obese, directly contributing to increased risk of developing diabetes, hypertension, and cardiovascular disease [2]. While a myriad of strategies exist to combat the downstream complications of obesity, little progress has been made in preventing the onset and development of these clinical manifestations. GLP-1 agonists have revolutionized the medical management of obesity, yet the drugs remain cost-prohibitive for the vast majority of patients, and long-term effects of these compounds have yet to be discovered. A significant gap in our knowledge is a complete understanding of the underlying mechanisms by which obesity causes cardiovascular disease, and efforts to break the inextricable link between the two have proven thus far unfruitful.

Obesity is a chronically inflamed state in which overproduction of reactive oxygen species (ROS) predominates, especially within the vasculature [3,4,5]. We and others have consistently demonstrated that ROS overproduction by NADPH oxidases is a central mechanism by which obesity causes vascular dysfunction [6,7]. Overproduction of superoxide leads to lipid peroxidation, protein dysfunction, and DNA damage in both the vascular endothelium and smooth muscle, severely inhibiting vasodilation [8]. Additionally, superoxide reacts with nitric oxide to produce peroxynitrite, further decreasing the bioavailability of nitric oxide and hastening a positive feedback cycle of ROS-induced cellular damage and decreased vasodilatory capacity [9,10]. This vasoconstrictive phenotype contributes to the development of endothelial dysfunction, a precursor and indeed predictor of downstream pathologies such as hypertension and atherosclerosis [11]. Metabolic regulation of ROS producing enzymes, thought to be an evolutionary consequence of increased aerobic metabolism, has been studied extensively, yet no therapeutic has proven effective in ameliorating ROS overproduction and downstream cardiovascular consequences [12,13].

While overproduction of ROS is central feature of obesity, not all ROS-producing enzymes are created equal. Indeed, ROS signaling is crucial for normal cardiovascular physiology; the knockdown of certain oxidative enzymes has shown to be deleterious to cardiovascular, metabolic, and renal health [14,15]. Canonically, NOX1 has been largely identified as a major source of deleterious superoxide production, whereas NOX4-mediated production of hydrogen peroxide has been shown to be cardio- and renal-protective [16]. NOX1 shares much of its structure with NOX2, the primary ROS producing enzyme in phagocytes. NOX1 is a six-pass transmembrane protein that requires several activating proteins for its proper function, including NOX organizer (NOXO1) and NOX activator (NOXA1) [17,18]. In addition to being regulated at the level of assembly, NOX1 is also subject to epigenetic regulation by histone deacetlyases (HDAC) 1 and 2 [19]. In the obese state, the normal physiologic degrees of NOX activation wax and wane, leading to a disproportionate concentration of free radicals which reduce nitric oxide bioavailability and propagate cellular death [20].

While scavenging of ROS with antioxidants has been extensively studied, no major study has yielded fruitful or clinically relevant results. Recent studies have sought to develop NOX inhibitors to halt overproduction of superoxide, as basal levels are required for normal physiologic processes and cell signaling [21]. The more recent development of “naxib”, or NOX inhibiting antibodies has ushered in a new focus on specific NOX isoform inhibitors, as previous pharmacologic inhibitors were selective across multiple isoforms. For example, GKT137831 or setanaxib has been demonstrated to inhibit both NOX1 and NOX4 and is currently in clinical trials for multiple cardiovascular diseases such as atherosclerosis and pulmonary hypertension [22,23]. ML090 has been demonstrated to be selective for NOX5 over NOX1 in humans, but since mice lack expression of NOX5, preclinical studies have proven uniquely difficult [24]. GKT771 is a NOX1 specific inhibitor that has been studied in the context of tumor angiogenesis, however its effect on other pathophysiologic mechanisms mediated by NOX1 remains an active area of exploration [25].

Herein, we tested the hypothesis that NOX1 is a major contributor to hypertension in obesity, and that pharmacological inhibition of NOX1 is a potential mechanism whereby initial oxidative insult to the endothelium may be abrogated. Our data demonstrate that NOX1 deletion ameliorates hypertension in obese mice independent of metabolic improvement and that the novel NOX1 inhibitor GKT771 is an attractive potential therapeutic for improving obesity-associated endothelial dysfunction central to the development of hypertension.

## 2. Materials and Methods

### 2.1. Data Availability

Data supporting this study may be made available by the corresponding author upon reasonable request.

### 2.2. Animal Studies

All animal experiments were conducted under Institutional Animal Care and Use Committee (IACUC) approval and in accordance with the NIH Guide for the Care and Use of Laboratory Animals. Mice heterozygous (*H_db_*) for mutation in the leptin receptor (Jackson Labs; strain no. 000697) were crossed with mice lacking NOX1 (*W_nox_*_1_) (Jackson Labs; strain no. 018787) to generate *H_db_W_nox_*_1_. Thus, the experimental paradigm included generation of four groups of mice: the lean control *H_db_W_nox_*_1_ (*Leprdb*/+*.Nox*1+/*y*), the lean NOX1 KO *H_db_K_nox_*_1_ (*Leprdb*/+*.Nox*1-/*y*), the obese control *K_db_W_nox_*_1_ (*Leprdb/db.Nox*1+/*y*), and obese NOX1 KO *K_db_K_nox_*_1_. (*KdbKnox*1). Genotyping of mice was performed by isolating DNA from tail clippings with the primers listed in Supplementary Table S1. Male mice were sacrificed at 18–22 weeks of age after being thoroughly anesthetized in an induction chamber with 5% isoflurane at 1 L/min O_2_, then decapitated by guillotine.

### 2.3. Metabolic Phenotyping

Before euthanasia, mice were weighed and subjected to Nuclear Magnetic Resonance (NMR) in a Minispec Body Composition Analyzer (Bruker; Model no. LF90II Billerica, MA, USA) to determine whole-body fat and lean percentages. Mice were fasted for 4 h before euthanasia and heparinized blood was collected. Blood samples were analyzed for fasting blood glucose using a standard glucometer (AlphaTrak, Zoetis, Parsippany, NJ, USA) and for HbA1c using a multi-test A1CNow HbA1c system (PTS Diagnostics, Whitestown, IN, USA). Additionally, a cohort of mice were monitored in a metabolic cage, for measurement of food and water intake, as well as urine output. Mice were allowed to acclimate to the metabolic cages for 24 h, then monitored for 72 continuous hours for the purpose of data collection. Data collection occurred every 24 h and urine was analyzed using an EasyLyte Electrolyte Analyzer (Medica Corporation, Bedford, MA, USA). Excretion was calculated mol/L times L/day to obtain mol/day excretion rate.

### 2.4. Gene Expression

RNA was isolated from tissue samples using Direct-Zol RNA MiniPrep Plus (Zymo; Lot no. ZRC204808, Irvine, CA, USA). cDNA was synthesized using OneScript cDNA Synthesis SuperMix (ABM; cat no. G452). qPCR was performed in a CFX-Connect Real-Time PCR Detection System (Bio-Rad, Hercules, CA, USA) utilizing BrightGreen Express 2X qPCR MasterMix-iCycler (ABM; cat no. MasterMix-EC, Vancouver, BC, Canada). Products were amplified at 95 °C for 3 min followed by cycles of 95 °C for 15 s, 58.5 °C for 15 s, and 72 °C for 15 s for 40 cycles. Expression fold change was calculated using the 2^−ΔΔCT^ method normalized to GAPDH as an internal control. Primer sequences are found in Supplementary Table S2.

### 2.5. Pressure Myography

Second and third order mesenteric arteries were dissected from intestines in ice-cold Krebs solution (118 mM NaCl, 25 mM NaHCO_3_, 11.1 mM d-glucose, 4.71 mM KCl, 2.56 mM CaCl_2_, 1.13 mM NaH_2_PO_4_, 7 mMMgCl_2_). Dissected arteries were trimmed free from adipose tissue, cannulated, and secured on glass pipettes in 10 mL of Krebs solution in a single vessel chamber (Living Systems Instrumentation, St. Albans, VT, USA). Microvessels were pressurized to physiological pressure of 60 mmHg with a Pressure Servo Controller (LSI; Model no. PS-200-S) and heated to physiological temperature of 37 °C with a Temperature Controller (LSI; Model no. TC-095). Vessels were allowed to equilibrate for 20 min, after which viability was assessed with 10 µL of saturated KCl (4.5 M). After three washes to ensure KCl removal, dose–response curves of acetylcholine, phenylephrine, and sodium nitroprusside (10^−9^–10^−4^ M) were generated sequentially, with three washes in between each of the curves. Myogenic tone was assessed at 20 mmHg increments (20–120 mmHg) by recording passive and outer diameters at each pressure. Tone was calculated as the % diameter of the prevailing pressure divided by the passive diameter at the same pressure. Vessel was washed three times in calcium-free Krebs solution to induce loss of tone and allowed to equilibrate for 60 min and reassessed at these incremental pressures. For inhibitor treatment, vessels were incubated in 10 uM GKT771 or vehicle for 30 min during initial equilibration.

### 2.6. Blood Pressure Telemetry

Telemetry experiments were performed as previously described [26]. Briefly, mice were anesthetized and implanted with DSI (St. Paul, MN, USA) telemetry units via the left carotid artery. Mice were allowed to recover for approximately 7 days, then blood pressure was recorded 24 h per day for 5 days. Care was taken to minimize handling of mice during data collection. MAP was calculated by adding DBP to one third of the difference in SBP and DBP.

### 2.7. Statistical Analyses

Differences between groups were assessed using a Student’s *t*-test or One-way ANOVA with Tukey’s Correction where appropriate. All analyses were performed with GraphPad Prism 9.1 (GraphPad Software Inc., San Diego, CA, USA). Data are expressed as mean ± standard error of the mean. *p* ≤ 0.05 was used as criteria for significance. * = *p* < 0.05, ns = not significant.

## 3. Results

### 3.1. Obesity-Induced Metabolic Derangement Persists Despite NOX1 Deletion

**Figure 1:**
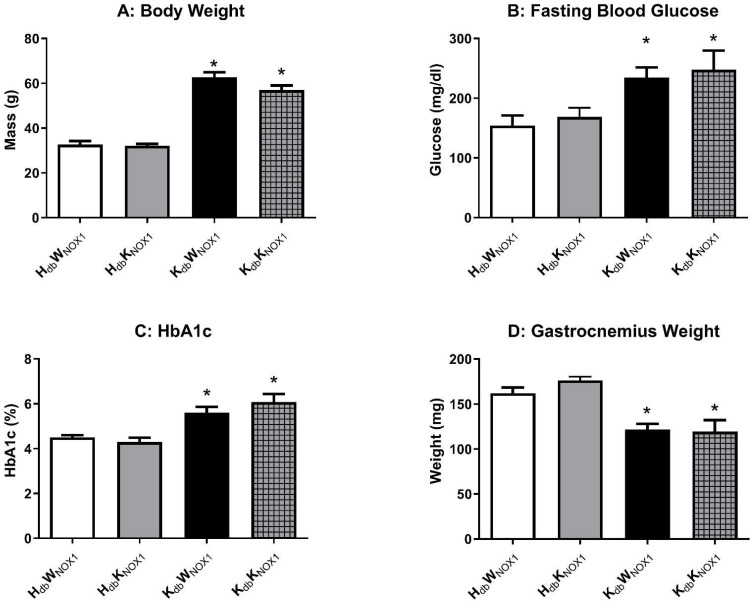
NOX1 deletion does not alter obesity-derived whole body metabolic derangement. Body weight (**A**) is unchanged intra-group, with the obese groups demonstrating a significant and marked increased in body weight. Further, fasting glucose (**B**) and Hba1c (**C**) are significantly elevated in the obese groups compared to lean controls. NOX1 deletion does not alter muscle mass (**D**) as gastrocnemius weight is significantly atrophied in both obese groups. *n* ≥ 5 for all groups. * *p* ≤ 0.05 versus *H_db_W_NOX_*_1_ controls.

In order to isolate the vascular role of NOX1 independent of improvements in metabolism, whole-body and tissue metabolic phenotyping of lean and obese NOX1 knockout mice was undertaken. NOX1 knockout mice were crossed with heterozygous leptin receptor-deficient mice to produce lean (HW), lean knockout (HK), obese (KW) and obese knockout (KK) mice. NOX1 deletion did not significantly alter body weight (Figure 1A), fasting blood glucose (FBG) (Figure 1B), HbA1c (Figure 1C) or skeletal muscle mass as measured by gastrocnemius weight (Figure 1D) in both lean and obese mice. NOX1 deletion did not alter key variables (body weight, muscle mass) in either lean or obese mice compared to their respective controls. Further, both obese groups had increased body weight, fasting glucose, and HbA1c as previously described, and also displayed marked sarcopenia compared to lean controls [27]. As shown in Table 1, obese mice were hyperlipidemic, hyperinsulinemic and had higher levels of circulating inflammatory cytokines independent of the presence or absence of NOX1. These data indicate that NOX1 deletion has no impact on the gross anatomic features of obese *db*/*db* mice, nor can changes in cardiovascular function be explained by gross changes in metabolism.

### 3.2. NOX1 Deletion Abrogates Obesity-Induced Hypertension

**Figure 2:**
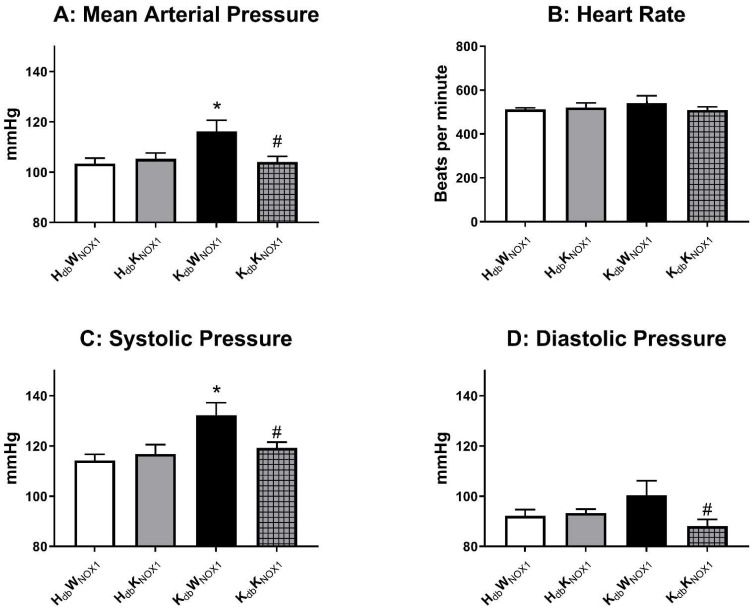
NOX1 deletion prevents obesity-induced hypertension. Figure 2 is the 5 day average of blood pressure indices recorded using in vivo radiotelemetry. Mean arterial pressure (**A**) is significantly elevated in obese control (*K_db_W_NOX_*_1_) compared to the other groups. Heart rate (**B**) is unchanged between the four groups. Systolic (**C**) and diastolic pressure (**D**) show similar increases in the obese control. *n* = 5 for lean groups and 7–9 for obese groups. * *p* ≤ 0.05 versus *H_db_W_NOX_*_1_ controls. # *p* ≤ 0.05 versus *K_db_W_NOX_*_1_.

In order to critically test the hypothesis that NOX1 is a major driver of hypertension in obesity, lean and obese mice with and without NOX1 deletion were subjected to in vivo radiotelemetry for assessment of blood pressure. As previously demonstrated, the obese control mice exhibited marked hypertension, which was ameliorated by whole-body NOX1 deletion (Figure 2A). NOX1 deletion, however, had no appreciable effect on heart rate (Figure 2B). Both systolic (Figure 2C) and diastolic (Figure 2D) pressures were elevated in obese mice and were likewise rescued by NOX1 deletion. These data indicate that NOX1 is indeed a major driver of hypertension in murine obesity, and deletion improves blood pressure independent of heart rate.

### 3.3. NOX1 Deletion Improves Renal Function in Obesity

**Figure 3:**
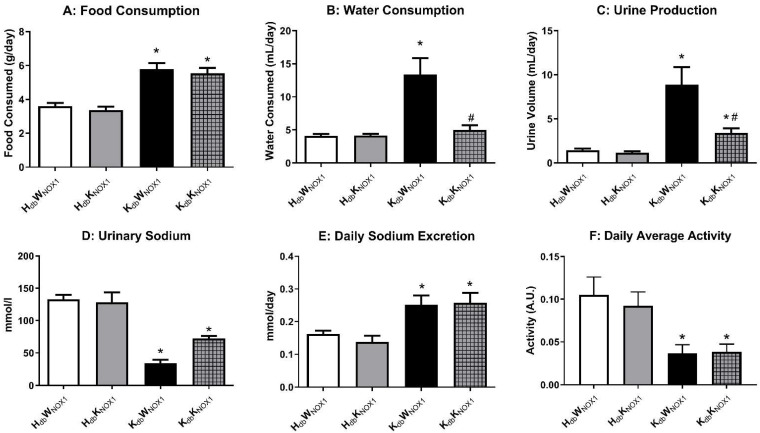
NOX1 deletion improves renal dynamics in the obese mouse. Metabolic cages were used to examine food and fluid intake in all groups. Food intake (**A**) was similar intra-group, albeit significantly elevated in the obese mice. Water consumption (**B**) and urine production (**C**) show significant polydipsia and polyuria in the obese control (*K_db_W_NOX_*_1_). Daily sodium balance is maintained (**D**), as sodium excretion (**E**) remains similar intra-group and elevated in the obese groups. *n* ≥ 8 for all groups. Obese mice exhibited lesser overall activity (**F**), which was likewise unchanged intra-group. * *p* ≤ 0.05 versus *H_db_W_NOX_*_1_ controls. # *p* ≤ 0.05 versus *K_db_W_NOX_*_1_.

As NOX1 deletion rescued blood pressure in obese control mice to that of healthy lean controls, we next turned to the role of NOX1 in the kidney. Mice underwent plasma metabolic phenotyping in order to determine if NOX1 deletion affected renal dynamics. As previously demonstrated, *db*/*db* mice consumed significantly more food compared to their lean counterparts, which was unaffected by NOX1 deletion (Figure 3A) [28]. However, NOX1 deletion significantly decreased total water consumption (Figure 3B) as well as total urine production (Figure 3C) in obese mice compared to healthy lean controls. Urinalysis revealed increased sodium excretion in obese NOX1 knockout mice as compared to obese mice (Figure 3D) but was unchanged when normalized to daily urine output (Figure 3E). Coupled with no change in daily activity (Figure 3F), we concluded that NOX1 deletion may lower blood pressure through alterations in renal sodium handling. Urinary potassium (Figure S1A,B) and chloride (Figure S1C,D) were similarly reduced in obesity and ameliorated by NOX1 deletion, with no change intra-group when normalized to daily output.

**Figure 4:**
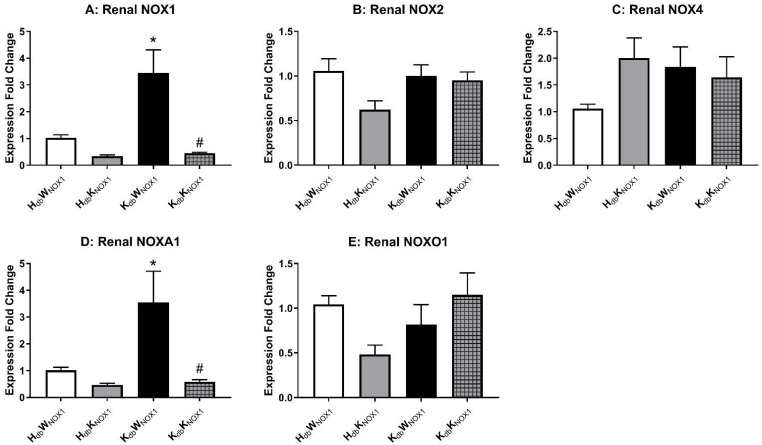
NOX1 deletion prevents obesity-derived increases in renal NOX1. Relevant NOX isoforms were examined in total renal homogenates. Panel (**A**) shows significant elevation of expression of NOX1 in the obese control (*K_db_W_NOX_*_1_), with no significant change in NOX2 (**B**) or NOX 4 (**C**) expression. Additionally, NADPH oxidase activator 1 (NOXA1, (**D**)) is also upregulated in the obese control, with no significant changes in NOXO1 (**E**) expression. *n* = 5–6 for all groups. * *p* ≤ 0.05 versus *H_db_W_NOX_*_1_ controls. # *p* ≤ 0.05 versus *K_db_W_NOX_*_1_.

Relevant NOX transcripts were measured in total kidney homogenates to determine if any compensatory upregulation of other ROS-producing enzymes occurs in the state of NOX1 deletion (Figure 4A). NOX 2 (Figure 4B) and NOX4 (Figure 4C) mRNA were not significantly increased in obese mice and were not altered by NOX1 deletion. However, transcripts of NOX activator (NOXA1) were significantly increased in obesity and were normalized by NOX1 deletion (Figure 4D). NOX organizer (NOXO1) was however unchanged among groups (Figure 4E). Therefore, while no compensatory upregulation of other NOX isoforms seems to occur in the kidney, NOXA1 expression mirrors that of NOX1 in the kidney of the obese mouse.

### 3.4. Pharmacologic Inhibition of NOX1 Rescues Microvascular Endothelial Function

**Figure 5:**
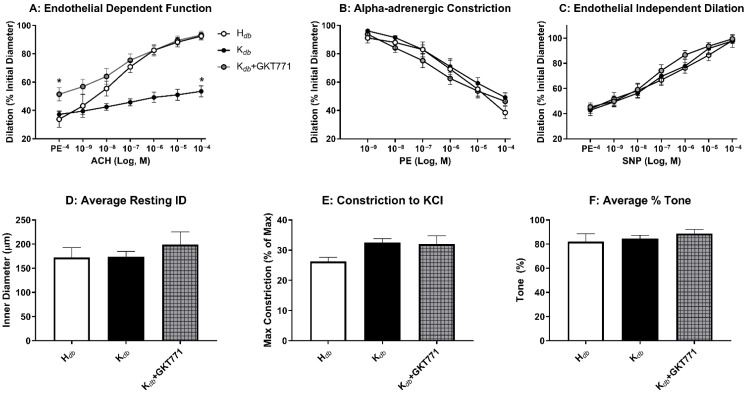
Pharmacologic inhibition of NOX1 with GKT771 rescues endothelial function in obese mice. Treatment with GKT771 rescued microvascular endothelial function in obese mice (**A**), with no appreciable effect on alpha-adrenergic constriction (**B**) or endothelial-independent vasodilation (**C**). Pharmacologic treatment did not alter resting internal diameter (**D**), maximum constriction to saturated KCl (**E**), or vascular tone (**F**). *n* = 5–6 for all groups. * *p* ≤ 0.05 versus *H_db_W_NOX_*_1_ controls.

We have previously demonstrated that NOX1 deletion preserves microvascular dysfunction in obesity, however pharmacologic approaches targeting NOX1 have been thus far elusive. We employed the novel NOX1-specific inhibitor GKT771 in an attempt to critically test the hypothesis that pharmacologic inhibition of NOX1 is sufficient to improve endothelial function in obese mice. In vessels treated with GKT771, obese mice had complete rescue of microvascular endothelial function to that of healthy controls in response to the eNOS-dependent vasodilator acetylcholine (Figure 5A). GKT771 treatment had no effect on alpha-adrenergic vasoconstriction (Figure 5B) or endothelium-independent vasodilation (Figure 5C). Resting internal diameter was constant amongst groups (Figure 5D), as was maximum constriction (Figure 5E), and percent tone (Figure 5F). These data demonstrate that GKT771 is a potent inhibitor of NOX1 that improves vasodilation in obese mice and presents a novel therapeutic approach to mitigating endothelial dysfunction, which is known to be a key driver of hypertension in obesity.

## 4. Discussion

In this study, we provide compelling evidence that NOX1 plays a central role in obesity-induced hypertension and endothelial dysfunction, demonstrating that genetic deletion of NOX1 improves vascular health and reduces blood pressure in obese mice. Furthermore, NOX1 upregulation can be targeted with pharmacologic inhibition with GKT771, which restored endothelial function in the microvasculature, suggesting that NOX1 inhibitors could represent a promising therapeutic class for mitigating obesity-associated cardiovascular disease. These findings concur both with our previous work that implicates NOX1 as the main driver of endothelial dysfunction in obesity and with other studies that point to NOX1-derived ROS overproduction as a hallmark of obesity-associated vascular inflammation [6,7]. Furthermore, NOX1 activation has been linked to other cardiovascular processes such as atherosclerosis and vascular remodeling, as well as diabetes and non-alcoholic hepatic steatosis, demonstrating its central role in the development of cardiometabolic disease [28,29,30].

The role of the NOX family of isoforms (1, 2, and 4) in renal function remains unclear in the literature. The dominant isoforms upregulated in pathology appear to be NOX2 and NOX4, but NOX1 has been noted to be specifically upregulated in the renal cortex in kidney disease [6,31]. An additional challenge has been that many studies evaluating the role of the NOX isoforms use systemic treatment of NOX1/4 dual inhibitors, rendering the identification of a specific renal isoform challenging. Further, the rodent models (similar to the *db*/*db* model we used) have a combination of hypertension and diabetes, progressive diseases that both contribute to endothelial and renal dysfunction [32]. This study is not able to dissect away a specific role for renal NOX1 due to the global tissue knockout but, given the improvements in blood pressure (with concurrent diabetic phenotype) and endothelial function, it does suggest protection against hypertension-induced renal damage.

In our work, GKT771 rescued endothelial function in obese mice by ameliorating overproduction of ROS by NOX1. Previous studies indicate that GKT771 has high specificity for NOX1 over other isoforms but may have some tangible effect on NOX4 in vivo [33]. Additionally, a broader understanding of the molecular pathways downstream of NOX1, such as its interaction with nitric oxide signaling or its role in inflammatory cytokine production, will be critical in evaluating the therapeutic potential of GKT compounds [34,35]. For example, studies examining the effects of NOX1 inhibition combined with existing antihypertensive therapies, could provide valuable insight into how NOX1 inhibition fits within the broader context of managing obesity-induced hypertension and related comorbidities [36,37]. Further, NOX1 inhibition could be a potential therapy to rescue obesity-associated endothelial dysfunction before the onset of hypertension, aiming to prevent downstream cardiovascular complications before they manifest [38]. As previously discussed, the role of NOX5 in the development of obesity-associated cardiovascular disease is incompletely understood; however, NOX5 is not expressed in mice—the species used in this study. Our data strongly indicate that NOX1 inhibition is sufficient to ameliorate endothelial dysfunction and hypertension in a preclinical murine model of obesity.

Caveats and limitations: We would be remiss if we did not state the limitations of the current work. First, these experiments are conducted in mice. While obese mice replicate many aspects of human disease, there may be unique aspects of human physiology that also contribute, such as the existence of NOX5. We also recognize the potential caveats of using genetic models, when humans will ultimately need pharmacotherapy. Current long-term in vivo studies with compounds such as GKT771 are cost-prohibitive but it is hoped that the current study will incentivize the exploration of such avenues in the future.

Taken together, our data strengthen the growing body of evidence that NOX1 underlies the development of vascular dysfunction in obesity and demonstrate that NOX1 deletion and inhibition improve indices of vascular health such as endothelial function and blood pressure. Pharmacologic inhibition with GKT771 provides an attractive field of study for the treatment and perhaps prevention of downstream cardiovascular complications.

## Figures and Tables

**Table 1:** Metabolic phenotyping of lean and obese mice with and without NOX1. *n* = 10 per group and * = *p* <0.05 vs. lean controls (*H_db_W_NOX_*).

	*H_db_W_NOX_*	*H_db_K_NOX_*	*K_db_W_NOX_*	*K_db_K_NOX_*
Cholesterol (mg/dL)	43 ± 3	39 ± 4	209 ± 13 *	207 ± 13 *
Triglycerides (mg/dL)	27 ± 1.5	28 ± 4	104 ± 5 *	119 ± 10 *
Insulin (ng/mL)	0.08 ± 0.01	0.07 ± 0.001	2.7 ± 0.3 *	1.9 ± 0.03 *
NEFA (mEq/mL)	1.5 ± 0.04	1.68 ± 0.07	5.7 ± 0.2 *	5.2 ± 0.3 *
TNF-a (pg/mL)	58 ± 5	64 ± 5	119 ± 17 *	98 ± 10 *

## Data Availability

Data is available from the corresponding author upon reasonable request.

## Supplementary Materials

The following supporting information can be downloaded at: https://www.mdpi.com/article/10.3390/antiox15010060/s1, Table S1. Primer sequences for genotyping; Table S2. Primer sequences for qPCR; Figure S1.

## References

[1] Cawley J., Biener A., Meyerhoefer C., Ding Y., Zvenyach T., Smolarz B.G., Ramasamy A. (2021). Direct medical costs of obesity in the United States and the most populous states. J. Manag. Care Spec. Pharm..

[2] Emmerich S.D., Fryar C.D., Stierman B., Ogden C.L. (2024). Obesity and Severe Obesity Prevalence in Adults: United States, August 2021–August 2023. NCHS Data Brief.

[3] Manna P., Jain S.K. (2015). Obesity, Oxidative Stress, Adipose Tissue Dysfunction, and the Associated Health Risks: Causes and Therapeutic Strategies. Metab. Syndr. Relat. Disord..

[4] Zhou Y., Li H., Xia N. (2021). The Interplay Between Adipose Tissue and Vasculature: Role of Oxidative Stress in Obesity. Front. Cardiovasc. Med..

[5] Nunan E., Wright C.L., Semola O.A., Subramanian M., Balasubramanian P., Lovern P.C., Fancher I.S., Butcher J.T. (2022). Obesity as a premature aging phenotype—Implications for sarcopenic obesity. Geroscience.

[6] Muñoz M., López-Oliva M.E., Rodríguez C., Martínez M.P., Sáenz-Medina J., Sánchez A., Climent B., Benedito S., García-Sacristán A., Rivera L. (2020). Differential contribution of Nox1, Nox2 and Nox4 to kidney vascular oxidative stress and endothelial dysfunction in obesity. Redox Biol..

[7] Thompson J.A., Larion S., Mintz J.D., Belin de Chantemèle E.J., Fulton D.J., Stepp D.W. (2017). Genetic Deletion of NADPH Oxidase 1 Rescues Microvascular Function in Mice With Metabolic Disease. Circ. Res..

[8] Didion S.P., Ryan M.J., Didion L.A., Fegan P.E., Sigmund C.D., Faraci F.M. (2002). Increased superoxide and vascular dysfunction in CuZnSOD-deficient mice. Circ. Res..

[9] van der Loo B., Labugger R., Skepper J.N., Bachschmid M., Kilo J., Powell J.M., Palacios-Callender M., Erusalimsky J.D., Quaschning T., Malinski T. (2000). Enhanced peroxynitrite formation is associated with vascular aging. J. Exp. Med..

[10] Pacher P., Szabó C. (2006). Role of peroxynitrite in the pathogenesis of cardiovascular complications of diabetes. Curr. Opin. Pharmacol..

[11] Gallo G., Volpe M., Savoia C. (2022). Endothelial Dysfunction in Hypertension: Current Concepts and Clinical Implications. Front. Med..

[12] Padgett C.A., Bátori R.K., Speese A.C., Rosewater C.L., Bush W.B., Derella C.C., Haigh S.B., Sellers H.G., Corley Z.L., West M.A. (2023). Galectin-3 Mediates Vascular Dysfunction in Obesity by Regulating NADPH Oxidase 1. Arterioscler. Thromb. Vasc. Biol..

[13] Pecchillo Cimmino T., Ammendola R., Cattaneo F., Esposito G. (2023). NOX Dependent ROS Generation and Cell Metabolism. Int. J. Mol. Sci..

[14] Santillo M., Colantuoni A., Mondola P., Guida B., Damiano S. (2015). NOX signaling in molecular cardiovascular mechanisms involved in the blood pressure homeostasis. Front. Physiol..

[15] Sirker A., Zhang M., Shah A.M. (2011). NADPH oxidases in cardiovascular disease: Insights from in vivo models and clinical studies. Basic Res. Cardiol..

[16] Schröder K., Zhang M., Benkhoff S., Mieth A., Pliquett R., Kosowski J., Kruse C., Luedike P., Michaelis U.R., Weissmann N. (2012). Nox4 is a protective reactive oxygen species generating vascular NADPH oxidase. Circ. Res..

[17] Helmcke I., Heumüller S., Tikkanen R., Schröder K., Brandes R.P. (2009). Identification of structural elements in Nox1 and Nox4 controlling localization and activity. Antioxid. Redox Signal..

[18] Dutta S., Rittinger K. (2010). Regulation of NOXO1 activity through reversible interactions with p22 and NOXA1. PLoS ONE.

[19] Manea S.A., Antonescu M.L., Fenyo I.M., Raicu M., Simionescu M., Manea A. (2018). Epigenetic regulation of vascular NADPH oxidase expression and reactive oxygen species production by histone deacetylase-dependent mechanisms in experimental diabetes. Redox Biol..

[20] Savini I., Catani M.V., Evangelista D., Gasperi V., Avigliano L. (2013). Obesity-associated oxidative stress: Strategies finalized to improve redox state. Int. J. Mol. Sci..

[21] Brand M.D. (2020). Riding the tiger—Physiological and pathological effects of superoxide and hydrogen peroxide generated in the mitochondrial matrix. Crit. Rev. Biochem. Mol. Biol..

[22] Thannickal V.J., Jandeleit-Dahm K., Szyndralewiez C., Török N.J. (2023). Pre-clinical evidence of a dual NADPH oxidase 1/4 inhibitor (setanaxib) in liver, kidney and lung fibrosis. J. Cell. Mol. Med..

[23] Streeter J., Thiel W., Brieger K., Miller F.J. (2013). Opportunity nox: The future of NADPH oxidases as therapeutic targets in cardiovascular disease. Cardiovasc. Ther..

[24] Dao V.T., Elbatreek M.H., Altenhöfer S., Casas A.I., Pachado M.P., Neullens C.T., Knaus U.G., Schmidt H.H.H.W. (2020). Isoform-selective NADPH oxidase inhibitor panel for pharmacological target validation. Free Radic. Biol. Med..

[25] Stalin J., Garrido-Urbani S., Heitz F., Szyndralewiez C., Jemelin S., Coquoz O., Ruegg C., Imhof B.A. (2019). Inhibition of host NOX1 blocks tumor growth and enhances checkpoint inhibitor-based immunotherapy. Life Sci. Alliance.

[26] Butcher J.T., Ali M.I., Ma M.W., McCarthy C.G., Islam B.N., Fox L.G., Mintz J.D., Larion S., Fulton D.J., Stepp D.W. (2017). Effect of myostatin deletion on cardiac and microvascular function. Physiol. Rep..

[27] Padgett C.A., Butcher J.T., Haigh S.B., Speese A.C., Corley Z.L., Rosewater C.L., Sellers H.G., Larion S., Mintz J.D., Fulton D.J.R. (2022). Obesity Induces Disruption of Microvascular Endothelial Circadian Rhythm. Front. Physiol..

[28] Larion S., Padgett C.A., Mintz J.D., Thompson J.A., Butcher J.T., Belin de Chantemèle E.J., Haigh S., Khurana S., Fulton D.J., Stepp D.W. (2024). NADPH oxidase 1 promotes hepatic steatosis in obese mice and is abrogated by augmented skeletal muscle mass. Am. J. Physiol. Gastrointest. Liver Physiol..

[29] Sheehan A.L., Carrell S., Johnson B., Stanic B., Banfi B., Miller F.J. (2011). Role for Nox1 NADPH oxidase in atherosclerosis. Atherosclerosis.

[30] Iwata K., Ikami K., Matsuno K., Yamashita T., Shiba D., Ibi M., Matsumoto M., Katsuyama M., Cui W., Zhang J. (2014). Deficiency of NOX1/nicotinamide adenine dinucleotide phosphate, reduced form oxidase leads to pulmonary vascular remodeling. Arter. Thromb. Vasc. Biol..

[31] Zhu K., Kakehi T., Matsumoto M., Iwata K., Ibi M., Ohshima Y., Zhang J., Liu J., Wen X., Taye A. (2015). NADPH oxidase NOX1 is involved in activation of protein kinase C and premature senescence in early stage diabetic kidney. Free Radic. Biol. Med..

[32] Gorin Y., Cavaglieri R.C., Khazim K., Lee D.Y., Bruno F., Thakur S., Fanti P., Szyndralewiez C., Barnes J.L., Block K. (2015). Targeting NADPH oxidase with a novel dual Nox1/Nox4 inhibitor attenuates renal pathology in type 1 diabetes. Am. J. Physiol. Ren. Physiol..

[33] Cipriano A., Viviano M., Feoli A., Milite C., Sarno G., Castellano S., Sbardella G. (2023). NADPH Oxidases: From Molecular Mechanisms to Current Inhibitors. J. Med. Chem..

[34] Liang S., Ma H.Y., Zhong Z., Dhar D., Liu X., Xu J., Koyama Y., Nishio T., Karin D., Karin G. (2019). NADPH Oxidase 1 in Liver Macrophages Promotes Inflammation and Tumor Development in Mice. Gastroenterology.

[35] He H., Jiang T., Ding M., Zhu Y., Xu X., Huang Y., Yu W., Ou H. (2025). Nox1/PAK1 is required for angiotensin II-induced vascular inflammation and abdominal aortic aneurysm formation. Redox Biol..

[36] Park J.M., Do V.Q., Seo Y.S., Kim H.J., Nam J.H., Yin M.Z., Kim H.J., Kim S.J., Griendling K.K., Lee M.Y. (2022). NADPH Oxidase 1 Mediates Acute Blood Pressure Response to Angiotensin II by Contributing to Calcium Influx in Vascular Smooth Muscle Cells. Arter. Thromb. Vasc. Biol..

[37] Matsuno K., Yamada H., Iwata K., Jin D., Katsuyama M., Matsuki M., Takai S., Yamanishi K., Miyazaki M., Matsubara H. (2005). Nox1 is involved in angiotensin II-mediated hypertension: A study in Nox1-deficient mice. Circulation.

[38] Versari D., Daghini E., Virdis A., Ghiadoni L., Taddei S. (2009). Endothelial dysfunction as a target for prevention of cardiovascular disease. Diabetes Care.

