# Supplementary material for: NADPH Oxidase 1 Mediates Endothelial Dysfunction and Hypertension in a Murine Model of Obesity
Source: Antioxidants (Basel). 2026 Jan 1;15(1):60. doi: 10.3390/antiox15010060 (PMC12837265; doi:10.3390/antiox15010060)
Supplement: Supplementary file 1 [file antioxidants-15-00060-s001.zip › antioxidants-3617014-supplementary.pdf]

Supplemental Materials

Table S1. Primer sequences for genotyping.

db MUT	Fwd	5'-CCCAACAGTCCATACAATATTAGAAGATTTTTACATTTTGA-3'
	Rev	5'-GTCCAAACTGAACTACATCAAACCTAC-3'
NOX1 WT	Fwd	5'-TTGAAAGGTTGGGTTTAGCTG -3'
	Rev	5'-AAATGGAACCCTTGGAGCA -3'
NOX1 MUT	Fwd	5'-TTGAAAGGTTGGGTTTAGCTG -3'
	Rev	5'-CGGTCCTCAAGAAGGAATC -3'

Table S2. Primer sequences for qPCR.

NOX1	Fwd	5'-CATGGCCTGGGTGGGATTGT-3'
	Rev	5'-TGGGAGCGATAAAAGCGAAGGA-3'
NOX2	Fwd	5'-TGTGGTTGGGGCTGAATGTC-3'
	Rev	5'-CTGAGAAAGGAGAGCAGATTTCG-3'
NOX4	Fwd	5'-AATGTTGGGCCTAGGATTGT-3'
	Rev	5'-TTCACTGAAAAGTTGAGGGC-3'
NOXA1	Fwd	5'- ACGGTGGATGTTCTGTGTGA -3'
	Rev	5'- AAGCATGGCTTCCACATAGG-3'
NOXO1	Fwd	5'- ACACGTCGGGGGCATACTGGTC -3'
	Rev	5'- GGCTGCCTCTGGGTTGGGATA-3'
GAPDH	Fwd	5'-CCCTTAAGAGGGATGCTGCC-3'
	Rev	5'-TACGGCCAAATCCGTTCACA-3'

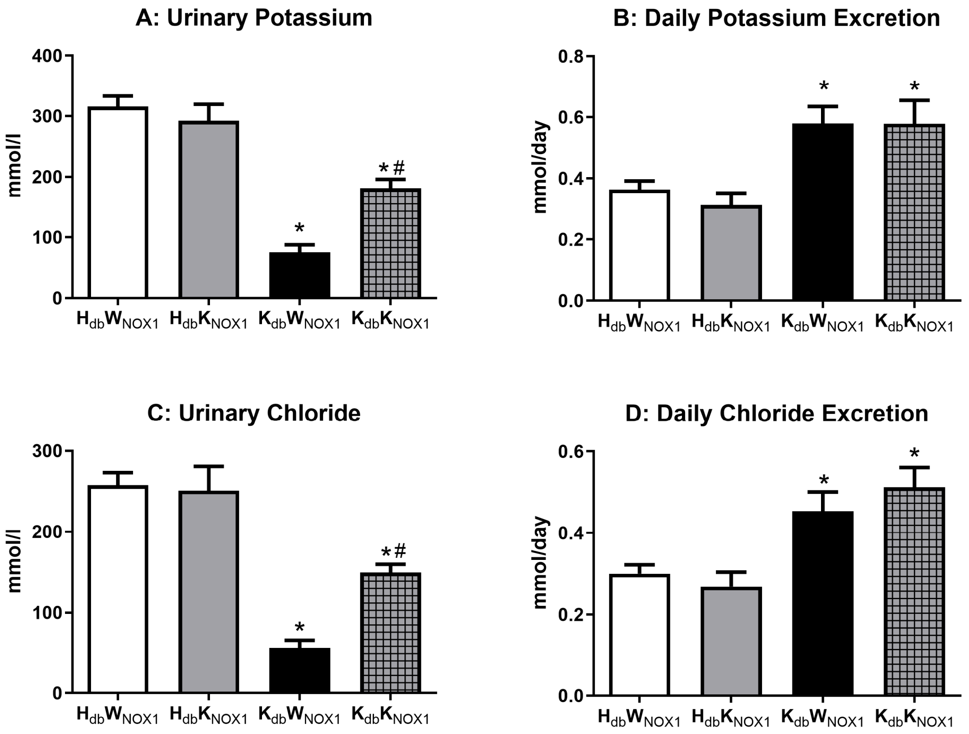

Figure S1. Excretion of secondary electrolytes in obese mice with and without NOX1. Above, urinary potassium concentration is depicted in **(A)** with total daily excretion in milligrams in **(B)**. Consistent with increased urinary volume in obesity, potassium concentration in diluted in NOX1 KO, lower pressure

reduces urine volume thus increase concentration. Accordingly, total excretion is similar with and without NOX1, though total potassium is increased in both obese mice as overall intake in similar and greater compared to leans. Below, urinary chloride concentration **(C)** and excretion **(D)** is depicted with a similar pattern and explanation. * = p<0.05 vs lean in the same strain, # = p<0.05 vs NOX1 intact in the same strain.
